# Visual recognition limitations in multimodal large language models: A comparative analysis of histological image interpretation

**DOI:** 10.1371/journal.pdig.0001306

**Published:** 2026-03-19

**Authors:** Volodymyr Mavrych, Einas M. Yousef, Ahmed Yaqinuddin, Aftab Ahmed Shaikh, Olena Bolgova

**Affiliations:** College of Medicine, Alfaisal University, Riyadh, Kingdom of Saudi Arabia; National University of Singapore, SINGAPORE

## Abstract

Multimodal large language models (LLMs) with image recognition capabilities have emerged as potential tools for medical image analysis, yet their performance in specialized domains like histology remains largely unexplored. The objective of this study was to systematically evaluate the performance of leading multimodal LLMs in histological image interpretation and assess their visual recognition capabilities. Four multimodal LLMs (GPT-4o, Claude Sonnet 4, Gemini 2.5 Flash, and Copilot) were evaluated using 144 histological images representing four tissue types (epithelial, connective, muscle, and nervous) at three magnification levels. Each image was assessed using three standardized questions: tissue identification, morphological features, and functional analysis. Three expert faculty members independently graded responses using a 4-point scale (1 = Poor to 4 = Excellent). Friedman tests, ICC, and post-hoc power analyses were performed with statistical significance set at p < .05. A clear performance hierarchy emerged with Gemini demonstrating superior performance (mean score: 3.35/4.00), significantly outperforming all other models. Copilot and GPT-4o tied for second place (both 2.76/4.00), while Claude showed the lowest performance (2.55/4.00). Performance varied across tissue types, with epithelial tissue showing the greatest inter-model variation. Inter-rater reliability was good across all models (ICC > 0.85), confirming assessment consistency. Post-hoc power analysis validated statistical significance for primary comparisons but indicated insufficient power to distinguish between the three lower-performing models. Current multimodal LLMs exhibit significant limitations in visual recognition relative to text processing performance. The substantial cross-modal performance gaps reveal some constraints in visual processing architectures, though the underlying mechanisms require further investigation. These findings establish technical benchmarks for multimodal LLM development and highlight the need for specialized visual processing innovations in their imaging processes.

## Author summary

In our increasingly digital world, artificial intelligence (AI) systems are being developed to help medical professionals analyze medical images. Our research examines how well the latest AI systems interpret microscopic images of human tissues compared to their ability to answer written questions about the same topic. We tested four leading AI systems by asking them to identify tissues, describe features, and explain functions based on microscopic images. Three expert faculty members evaluated their responses. We discovered that while these AI systems excel at answering text-based medical questions (over 90% accuracy), they struggle significantly with interpreting images (51-80% accuracy). This performance gap varied between different AI systems and tissue types. Our findings reveal that current AI technology demonstrates substantial performance gaps in processing visual medical information despite having the necessary medical knowledge, suggesting opportunities for architectural improvements. This research provides important benchmarks for measuring progress in medical AI development and highlights challenges that must be addressed before these systems can be reliably used in medical education and practice.

## Introduction

The accurate interpretation of histological images is fundamental to medical diagnosis, research, and education, particularly in the field of pathology. Histopathology has long been considered the gold standard for disease diagnosis, requiring extensive expertise and experience to identify subtle morphological changes in tissues and cells that are characteristic of various pathological conditions [1]. Traditional histopathological analysis involves the visual examination of tissue samples under a microscope, where pathologists must recognize complex patterns and features to make diagnostic determinations. This process is not only time-consuming but also subject to inter-observer variability, creating challenges for consistent diagnosis and educational assessment [2].

In recent years, digital pathology has transformed the traditional practice of analyzing tissue under a microscope into a computer vision workflow through the adoption of whole-slide imaging, which allows pathologists to view and analyze microscopic images on a computer monitor [2]. This digitization of histopathology has opened new research pathways and insights in cancer prediction and prognosis, with a surge in deep learning and computer vision techniques for analyzing digital images [3]. Computational pathology, by leveraging artificial intelligence (AI) and machine learning (ML), has emerged as a promising field that can potentially assist pathologists in their workflow, reduce variability, and improve diagnostic accuracy [4].

The advent of large language models (LLMs) has further revolutionized the landscape of AI applications in healthcare. LLMs are sophisticated AI systems trained on vast amounts of text data, enabling them to understand and generate human-like text based on the input they receive. These models have demonstrated remarkable capabilities in various domains, including medicine, where they can process and analyze complex medical information to generate responses that rival human experts [2,3]. Recent advances have extended LLMs beyond text processing to include image analysis through multimodal architectures, making them potentially valuable tools for interpreting histological images [4].

Multimodal large language models combine LLMs’ natural language processing capabilities with computer vision techniques, allowing them to interpret both textual and visual information simultaneously [5]. This integration is particularly relevant for histopathology, where the ability to analyze visual patterns in tissue samples and communicate findings in natural language is essential for diagnosis, prognosis, and treatment planning [6]. Multimodal LLMs can potentially assist pathologists by providing automated analysis of histological images, extracting relevant features, and offering interpretable explanations of their findings [2].

Several studies have evaluated the performance of AI systems in various medical imaging domains, including radiology, dermatology, and ophthalmology [6–9]. These studies have shown promising results, with AI models sometimes achieving diagnostic accuracy comparable to human experts. For instance, convolutional neural networks, a type of deep learning model, have demonstrated excellent capabilities in tasks such as tumor detection, classification, and segmentation in medical images, with performance reaching or exceeding professional radiologists in some specific tasks [10]. However, the application of multimodal LLMs specifically to histopathology is still an emerging area of research with significant potential for advancement [11].

The integration of LLMs into medical education has been increasingly studied across various basic science disciplines, revealing patterns that reflect both the capabilities and limitations of these AI systems. Studies evaluating LLM performance in biochemistry, neuroscience, embryology, and gross anatomy have shown that these models can often answer complex medical questions with accuracy comparable to or sometimes exceeding that of medical students [12–14]. However, performance varies significantly across disciplines, with models performing better in knowledge-based domains than those requiring spatial reasoning and visualization [15]. This variation in performance extends to image interpretation, where LLMs typically demonstrate lower accuracy compared to their text-based capabilities [16].

The ability of LLMs to understand and process medical knowledge forms a crucial foundation for their application in image analysis. Many researchers found that while LLMs can serve as valuable adjuncts for instructional planning and assessment in medical education, expert review remains necessary to ensure content validity [15,17]. This suggests that despite their impressive capabilities in medical knowledge representation, LLMs may still face challenges in accurately interpreting and analyzing medical images, particularly in complex domains like histopathology [18].

The assessment of LLMs in histology education represents a promising frontier. Despite advances in multimodal LLMs showing promising results in medical image analysis, including radiology, dermatology, pathology, and orthopedic applications, there remains a limited systematic evaluation of LLMs specifically for histology education [19–21]. Most existing studies focus on diagnostic applications rather than educational utility, creating a knowledge gap regarding their effectiveness as teaching tools in medical education [22].

The integration of multimodal LLMs into medical education, particularly in histology, could potentially transform how students learn to identify and interpret tissue structures. Traditional histology education relies heavily on the expertise of instructors to guide students through the complex process of tissue identification and analysis with standardized, detailed rubrics developed for each question to ensure consistent evaluation criteria [23]. LLMs could provide personalized feedback, explanations, and assessments to support student learning, potentially enhancing the efficiency and effectiveness of medical education [24].

While previous studies have evaluated LLM performance on text-based medical questions [12–16] and general medical imaging tasks [6–9], this study provides the first systematic comparison of leading multimodal LLMs specifically for histological image interpretation in an educational context. Unlike prior work focusing on diagnostic applications [18], our research directly compares performance on histological content presented as images. Previous study established that LLMs achieve 90.3-92.0% accuracy on text-based histology course questions [25]; the current study evaluates their visual recognition capabilities, providing quantifiable metrics for the magnitude of the cross-modal performance gap.

This research project addresses the knowledge gap in evaluating current LLMs and their multimodal capabilities in analyzing histological images, specifically focusing on different tissue and cell identification. The study aims to assess existing LLMs’ accuracy, reliability, and educational potential when applied to histology image interpretation, ultimately determining their viability as educational tools in medical curricula. By systematically evaluating leading LLMs using histology image datasets, this research seeks to provide insights into the strengths and limitations of these models in histology image analysis and their potential integration into medical education.

## Materials and methods

### Study design

This study employed a cross-sectional, comparative evaluation design to systematically assess the performance of leading LLMs in medical image interpretation. Four leading multimodal LLMs with image recognition capabilities were selected based on their current market availability, documented multimodal capabilities, and public accessibility. These included GPT-4o (OpenAI, model version gpt-4o-2024-05-13), Claude Sonnet 4 (Anthropic, model version claude-sonnet-4–20240620), Gemini 2.5 Flash (Google, model version gemini-2.5 Flash-001), and Copilot (Microsoft, powered by GPT-4 Turbo, accessed via web interface). All models were accessed through their respective consumer-facing web interfaces. Inference parameters were set to platform defaults: temperature was not manually specified (platform-specific defaults varied), no reproducibility seeds were used, as this evaluation focused on the typical user experience rather than deterministic outputs, and response length was unconstrained. Maximum token limits followed each platform’s standard settings (approximately 4,000–8,000 tokens depending on platform). The research utilized a blinded evaluation protocol with multiple independent assessments to ensure objectivity and minimize bias. The study was designed as a benchmark comparison across different LLM platforms using standardized histological image datasets with predetermined ground truth annotations validated by expert histologists. Only images achieving 100% expert agreement were included in the evaluation dataset.

The histological image dataset was compiled from the existing slide collection at the Anatomy Department of College of Medicine at Alfaisal University. Four basic tissue types were included: epithelial, connective, muscle, and nervous tissues. Each tissue type was represented by 12 slides with 3 magnification levels per slide (40x, 100x, and 400x), yielding 144 total images. The majority of slides were stained with Hematoxylin and Eosin; special stains included silver staining, Aldehyde Fuchsin, Masson’s trichrome, Phosphotungstic Acid-Hematoxylin, Periodic Acid-Schiff, Cresyl Violet, and Toluidine blue for specific tissue characteristics. Connective tissue slides included loose areolar, dense regular and irregular, adipose, cartilage, and bone tissues. Epithelial slides included simple and stratified epithelia of various types (squamous, cuboidal, columnar) and glandular tissue. Muscular slides included skeletal, cardiac, and smooth muscle in various orientations. Nervous tissue slides included the spinal cord, ganglia (dorsal root, sympathetic, parasympathetic), and peripheral nerves. All images were captured at 1920 × 1440 pixels using standardized whole-slide imaging, saved as JPEGs, and quality-controlled for consistent focus and illumination. Only images achieving 100% expert agreement on ground truth were included.

### Data collection

A standardized evaluation protocol, consisting of three core questions for each histological image, was aligned with the established medical college educational framework. Consistent prompt templates were developed and validated with 3 questions addressed: Q1 Tissue/Organ Identification (“What tissue/organ is shown in [ ] histological section?”), Q2 Morphological Features (“Identify the marked by [ ] structure. What are its morphological features?”), and Q3 Functional Analysis (“What is the primary function of the indicated by [ ] structure?”).

All LLM responses were collected during June-July 2025. Testing sessions were conducted under controlled conditions with standardized parameters maintained across all platforms. Each LLM was evaluated on each slide with all three questions. The identical prompts were used to assess each model’s response consistency and reliability. Results were compiled into separate files for each tissue and LLM.

Expert evaluations were conducted using a 4-point scale where 1-Poor/Incorrect, 2-Fair/Partially correct, 3-Good/Correct, and 4-Excellent/Absolutely correct. Three faculty members holding professorial rank (Expert X, Expert Y, and Expert Z), each with more than 15 years of teaching experience, served as independent evaluators, graded responses independently without knowing LLM identity or other experts’ grades. Standardized, detailed rubrics were developed for each question to ensure consistent evaluation criteria (S1 File). 1,728 expert assessments were collected in total: 4 tissues x 12 slides (each with 3 images of different magnification levels given as a single set) x 3 questions x 4 LLMs x 3 experts.

### Statistical analysis

Performance metrics were calculated for each LLM, including overall accuracy rates by tissue type, response consistency measures across multiple trials, and inter-rater reliability assessments. The primary outcome measure was the correct tissue identification rate, while secondary outcome measures included the morphological feature accuracy score and the functional explanation completeness score.

Mean scores with standard errors and 95% confidence intervals were calculated for each LLM’s overall performance and for performance by tissue type and question category. Accuracy percentages reported in this study were calculated as the proportion of responses achieving correct or absolutely correct ratings. Specifically, responses scoring 3 (Good/Correct) or 4 (Excellent/Absolutely correct) were classified as accurate, while responses scoring 1 (Poor/Incorrect) or 2 (Fair/Partially correct) were classified as inaccurate. Accuracy was then calculated as: Accuracy (%) = [(number of responses scoring ≥3)/ (total number of responses)] × 100.

Comparative analysis employed the Friedman test (non-parametric equivalent of repeated-measures ANOVA) to assess performance differences across models, with post-hoc pairwise comparisons using appropriate critical difference values to determine statistical significance. Chi-square tests were used to compare categorical accuracy rates between LLMs.

Reliability and consistency assessment included calculation of Intraclass Correlation Coefficient (ICC) to assess agreement between the three expert evaluators (X, Y, and Z) who independently rated each model’s responses. ICC values were interpreted as follows: ICC > 0.9 = excellent reliability, 0.75-0.9 = good reliability, 0.5-0.75 = moderate reliability, and < 0.5 = poor reliability.

Effect sizes for model comparisons were calculated using Cohen’s d, where d < 0.2 = negligible effect, 0.2 ≤ d < 0.5 = small effect, 0.5 ≤ d < 0.8 = medium effect, and d ≥ 0.8 = large effect. Post-hoc power analysis was conducted at multiple significance levels (α = 0.05, α = 0.01, and α = 0.001) to verify the adequacy of the sample size and validate the statistical significance of findings.

All statistical analyses were performed using R version 4.3.0 (R Foundation for Statistical Computing, Vienna, Austria). Statistical significance was set at p < 0.05 for all analyses, with appropriate corrections applied for multiple comparisons.

## Results

The statistical analysis revealed a clear performance hierarchy among the evaluated LLMs. Gemini demonstrated superior performance with a mean score of 3.35/4.00, significantly outperforming all other models. Copilot and GPT-4o tied for second place with identical mean scores of 2.76/4.00. Claude showed the lowest performance with a mean score of 2.55/4.00 (Table 1).

**Table 1:** Overall performance of LLMs in histological image interpretation.

LLM	Mean Score ± SE	95% Confidence Interval	% Difference from Top LLM	Statistical Significance
Gemini	3.35 ± 0.05	3.25-3.46	–	–
Copilot	2.76 ± 0.06	2.64-2.89	-17.6%	0.465
GPT-4o	2.76 ± 0.06	2.64-2.88	-17.6%	0.691*
Claude	2.55 ± 0.07	2.41-2.68	-23.9%	0.622*

Mean scores based on a 4-point scale where 1 = Poor, 2 = Fair, 3 = Good, and 4 = Excellent. SE = Standard Error. % Difference shows percentage decrease compared to the top-performing model (Gemini). Statistical Significance shows mean rank difference from Friedman test, where values marked with * indicate statistically significant differences (p < 0.05).

**Fig 1:**
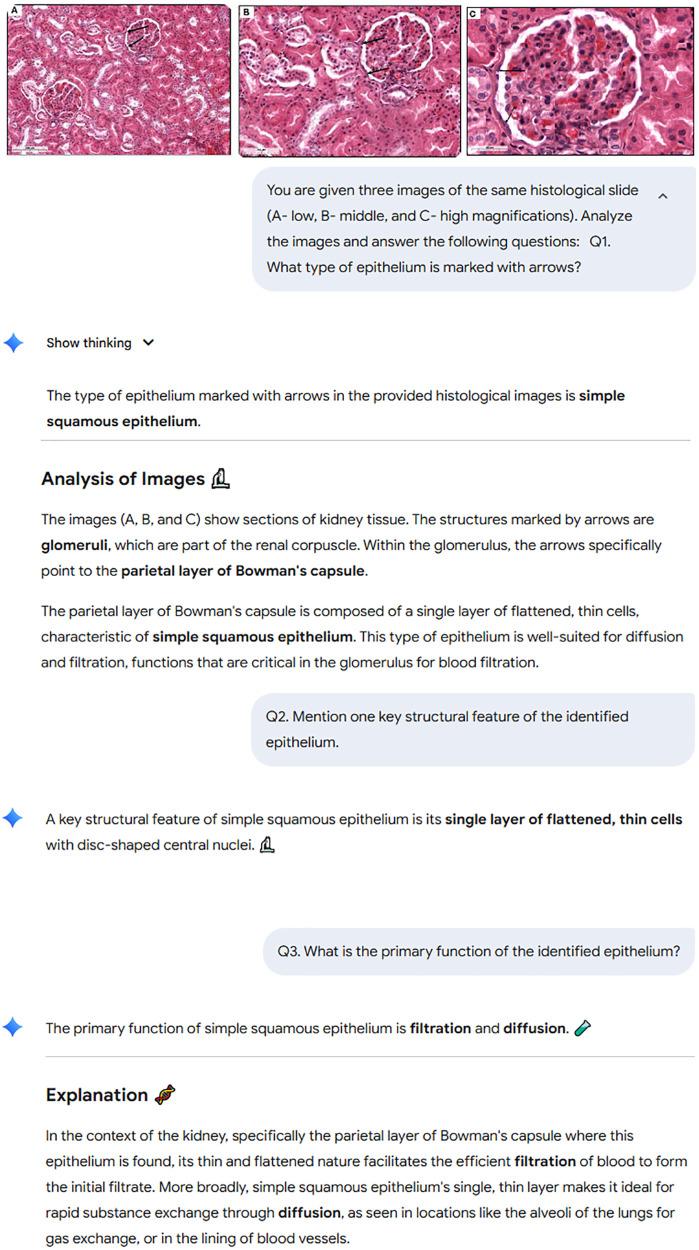
Screenshot of the histological images (A, B, C), prompts for Q1-Q3 and Gemini answers.

The example of Gemini answers the three questions regarding slide 1 from epithelial tissue section are shown on the screenshot below (Fig 1).

The distribution of expert scores across the four LLMs reveals distinctive performance patterns in histological image interpretation (Table 2). Gemini demonstrated the most consistent high-quality performance with 70.4% of responses rated as Excellent and only 15.3% rated as Poor, indicating reliable accuracy across tissue types and question categories. In contrast, Claude showed the most polarized performance profile with 46.5% Excellent ratings but also the highest Poor rating percentage (42.4%), suggesting significant inconsistency in its histological image analysis capabilities. Copilot and GPT-4o displayed intermediate patterns, with Copilot showing a bimodal distribution heavily weighted toward Excellent (49.8%) and Poor (32.2%) ratings, while GPT-4o exhibited more balanced performance with the highest proportion of Good ratings (21.5%) among all models.

**Table 2:** Distribution of expert scores by model (%).

LLM	Poor (1)	Fair (2)	Good (3)	Excellent (4)
Gemini	15.3%	4.6%	9.7%	70.4%
Copilot	32.2%	9.0%	9.0%	49.8%
GPT-4o	28.5%	8.6%	21.5%	41.4%
Claude	42.4%	6.9%	4.2%	46.5%

### Performance by tissue type and question type

**Fig 2:**
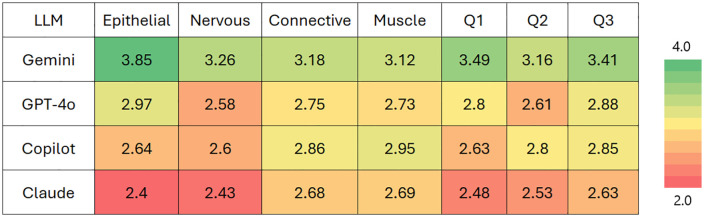
Heatmap of average LLM performance across different histological tissues recognition and questions asked. Tissue types include Epithelial, Nervous, Connective, and Muscle tissues. Question types include Tissue Identification (Q1), Morphological Features (Q2), and Functional Analysis (Q3). Numbers in cells indicate mean scores based on a 4-point scale (1 = Poor, 2 = Fair, 3 = Good, and 4 = Excellent answers).

Examination of performance across different tissue types and question types revealed distinctive patterns (Fig 2). Gemini consistently outperformed other models across all categories, with particularly strong results in epithelial tissue (3.85/4.00) and tissue identification questions (3.49/4.00). Claude generally scored lowest across most categories, with its weakest performance on epithelial tissue interpretation (2.40/4.00).

Epithelial tissue interpretation showed the greatest performance variation between models, while muscle tissue had relatively more consistent scores across LLMs. This suggests that epithelial tissue interpretation may be a more discriminating benchmark for evaluating LLM capabilities in histopathology.

The analysis across different question types reveals that Gemini maintained superior performance across all question categories. Question 1 (tissue identification) and Question 3 (functional analysis) showed greater performance differences between models than Question 2 (morphological features). These results suggest that certain aspects of histological image interpretation, particularly tissue identification and functional analysis, present different levels of challenge for current LLM architectures.

### Expert inter-rater reliability

Evaluation of inter-rater reliability among the three expert evaluators (X, Y, and Z) showed excellent consistency in their assessment of each LLM’s performance (Table 3). This reliability analysis measures the degree of agreement between the three histology experts who independently evaluated each model’s responses. Claude’s evaluations showed the highest expert agreement with an ICC of 0.989, followed by Copilot (ICC = 0.985), Gemini (ICC = 0.979), and GPT-4o (ICC = 0.945). Reliability was consistent across tissue types, with only minor variations. GPT-4o showed slightly lower expert agreement for connective tissue (ICC = 0.850), while all other tissue-specific ICCs were above 0.95.

**Table 3:** Expert inter-rater reliability in evaluating LLM performance.

Model	Overall ICC	Connective	Epithelial	Muscle	Nervous
Claude	0.989	0.990	0.990	0.987	0.991
Copilot	0.985	0.990	0.986	0.980	0.986
Gemini	0.979	0.987	0.969	0.966	0.985
GPT-4o	0.945	0.850	0.951	0.977	0.987

Intraclass Correlation Coefficient (ICC) values measure agreement among the three expert evaluators (X, Y, and Z) who rated each LLM’s performance. ICC > 0.9 = excellent reliability, 0.75-0.9 = good reliability, 0.5-0.75 = moderate reliability, and < 0.5 = poor reliability. All models demonstrated excellent overall reliability, indicating consistent evaluation across the three expert raters.

### Statistical significance differences between LLMs performances

The Friedman test indicated significant differences between LLMs performances (χ² = 25.06, df = 3, p < 0.001). Post-hoc pairwise comparisons showed significant differences between Gemini vs. Claude (difference = 0.622, critical difference = 0.552) and Gemini vs. GPT-4o (difference = 0.691, critical difference = 0.552). Non-significant differences were found between Copilot vs. Gemini (difference = 0.465), Copilot vs. Claude (difference = 0.156), Copilot vs. GPT-4o (difference = 0.226), and Claude vs. GPT-4o (difference = 0.069) (Table 4).

**Table 4:** Pairwise comparisons of LLMs using Friedman test with post-hoc analysis.

Comparison	Mean Rank Difference	Critical Difference	Significant?
Gemini vs. GPT-4o	0.691	0.552	Yes
Gemini vs. Claude	0.622	0.552	Yes
Copilot vs. Gemini	0.465	0.552	No
Copilot vs. GPT-4o	0.226	0.552	No
Copilot vs. Claude	0.156	0.552	No
Claude vs. GPT-4o	0.069	0.552	No

Results from Friedman test (non-parametric equivalent of repeated-measures ANOVA) with post-hoc comparisons. Mean rank differences greater than the critical difference (0.552) indicate statistically significant differences between LLMs (p < 0.05).

### Post-hoc power analysis

To verify the adequacy of the sample size and validate the statistical significance of our findings, post-hoc power analysis was conducted for the primary statistical tests used in this study (Table 5).

**Table 5:** Overall study power analysis.

Comparison	Effect Size (d)	Mean Difference	Power at α = 0.05	Power at α = 0.01	Power at α = 0.001
Gemini vs Claude	0.629	0.803	1.000	1.000	1.000
Gemini vs Copilot	0.476	0.588	1.000	1.000	0.527
Gemini vs GPT-4o	0.499	0.593	1.000	1.000	0.668
Copilot vs Claude	0.155	0.215	0.000*	0.000*	0.000*
Copilot vs GPT-4o	0.004	0.005	0.000*	0.000*	0.000*
Claude vs GPT-4o	0.157	0.211	0.000*	0.000*	0.000*

Cohen’s d, measuring the standardized difference between two means. Interpretations: d < 0.2 = negligible effect, 0.2 ≤ d < 0.5 = small effect, 0.5 ≤ d < 0.8 = medium effect, d ≥ 0.8 = large effect. Mean Difference: The absolute difference between the mean scores of the two models being compared (on a 4-point scale). Power: The probability of detecting a true effect at the specified significance level (α). Power ≥ 0.8 is generally considered adequate for statistical reliability. α: The significance level or probability of Type I error (false positive). *: Power values of 0.000 indicate insufficient statistical power to detect the effect at the given significance level.

The post-hoc power analysis reveals several key insights about the statistical robustness of the comparisons between LLMs in histological image interpretation:

- High-powered primary comparisons: The study demonstrated excellent statistical power (1.000) at both α = 0.05 and α = 0.01 levels for detecting differences between Gemini and all other models (Claude, Copilot, and GPT-4o). This indicates that the sample size (144 images with 3 expert raters) was more than adequate for these comparisons, and the observed performance differences are highly reliable.
- Strong effects even at stringent thresholds: For comparisons involving Gemini vs. other models, even at the most stringent significance level (α = 0.001), the power remained substantial (ranging from 0.527 for Gemini vs. Copilot to 1.000 for Gemini vs. Claude). This demonstrates robust statistical validity for the finding that Gemini significantly outperforms other LLMs in histological image interpretation.
- Insufficient power for secondary comparisons: The study had inadequate statistical power to detect the smaller differences between Copilot vs. Claude, Copilot vs. GPT-4o, and Claude vs. GPT-4o. The effect sizes for these comparisons were small (d = 0.004-0.157), suggesting that these models perform more similarly to each other. The nearly zero effect size between Copilot and GPT-4o (d = 0.004) indicates virtually identical performance levels.

The post-hoc power analysis confirms that while the study had more than sufficient statistical power to establish Gemini’s superior performance over other models, it could not reliably distinguish between the performances of the three lower-performing models. This suggests that from a practical perspective, Copilot, Claude, and GPT-4o may be considered similarly effective for histological image interpretation in educational settings, while Gemini represents a clearly superior option.

## Discussion

This evaluation of multimodal LLMs in histological image interpretation represents one of the first systematic assessments of current AI visual recognition capabilities in specialized medical imaging domains. Our findings reveal significant performance disparities among the evaluated models and, when compared with recent text-based histology assessments, expose fundamental limitations in current multimodal AI architectures for visual pattern recognition tasks.

### Visual recognition performance vs. Text processing capabilities

The performance hierarchy observed in histological image interpretation, with Gemini achieving 80.1% accuracy, followed by GPT-4o (63.0%), Copilot (58.8%), and Claude (50.7%), reveals substantial deficits when compared to the same models’ text-based performance on histology MCQs (S2 File). Recent comparative studies demonstrate that these same LLMs achieve remarkably higher accuracy in text-based histological assessments: Gemini (92.0%), Claude (91.5%), Copilot (91.0%), and GPT-4 (90.8%) [25]. This comparison reveals a visual recognition performance gap ranging from 11.9% (Gemini) to 40.8% (Claude), indicating fundamental architectural limitations in current multimodal systems.

The magnitude of these performance decrements suggests that visual processing pipelines in current LLMs are substantially less developed than their natural language processing capabilities. While text-based medical knowledge assessment achieves near-optimal performance (>90% across all models), visual interpretation of the same domain knowledge drops to poor-to-good levels (50.7-80.1%), highlighting the disparity between factual knowledge representation and visual pattern recognition in current AI architectures.

The differential performance patterns across models provide insights into varying visual recognition architectures and training methodologies. Gemini’s consistent superiority in both modalities (92.0% text vs. 80.1% visual) suggests more robust multimodal integration, with only an 11.9% performance decline when transitioning from text to visual processing. This relatively smaller gap indicates more effective visual feature extraction and cross-modal knowledge transfer capabilities.

In contrast, Claude demonstrates the most dramatic visual processing deficit, experiencing a 40.8% performance drop from text (91.5%) to visual interpretation (50.7%). This substantial degradation suggests potential limitations in Claude’s visual encoder architecture or training data composition for specialized medical imaging tasks. The stark contrast between Claude’s excellent text comprehension and poor visual recognition performance indicates that strong language modeling capabilities do not necessarily translate to effective visual analysis.

Copilot and GPT-4o showed intermediate visual processing capabilities with 32.2% and 27.8% performance gaps respectively, suggesting similar underlying architectural approaches to multimodal processing. Their comparable performance levels in both text and visual domains indicate potentially shared or similar visual processing frameworks [26].

### Tissue-specific visual recognition challenges

Analysis of tissue-specific performance patterns reveals consistent visual recognition challenges across different histological structures. Epithelial tissue interpretation showed the greatest inter-model variation (range: 2.40-3.85/4.00), suggesting that certain visual patterns may be more amenable to automated recognition than others. Interestingly, muscle tissue, which showed the lowest performance in text-based MCQs (76.0% average) [25], also demonstrated challenging visual recognition patterns across all models.

This cross-modal consistency in challenging tissue types indicates that certain histological concepts present fundamental recognition difficulties regardless of input modality [11]. However, the performance gaps are significantly more pronounced in visual tasks, suggesting that visual pattern complexity compounds the underlying conceptual challenges. The fact that topics achieving perfect text-based performance (Histological Methods, Blood and Hemopoiesis) showed substantially lower visual recognition accuracy indicates that factual knowledge about tissue characteristics does not automatically enable visual identification of those same features [25].

The substantial performance disparities between text and visual processing reveal critical insights about current multimodal AI architectures. The consistent pattern of visual underperformance across all models suggests systemic limitations in how visual information is processed, encoded, or integrated with pre-existing knowledge representations [27]. Current multimodal LLMs appear to excel at factual knowledge retrieval and reasoning but struggle with the spatial pattern recognition and morphological analysis required for histological image interpretation.

The bimodal distribution patterns observed in visual tasks, particularly for Claude and Copilot, indicate inconsistent visual processing capabilities that contrast sharply with the reliable text-based performance [28]. This suggests that visual processing pipelines may be less robust or more sensitive to input variations than text-processing components, leading to unpredictable performance on visually similar inputs.

### Visual feature extraction and pattern recognition limitations

The poor-to-good visual recognition performance levels (50.7-80.1%) compared to exceptional text performance (90.3-92.0%) highlight limitations in visual feature extraction capabilities. Current multimodal architectures appear unable to effectively translate detailed morphological features, such as cellular arrangement patterns, tissue architecture, and structural relationships into the same level of accurate interpretation achieved with textual descriptions of identical concepts.

The reliability analysis showing excellent inter-expert agreement (ICC > 0.945) validates that the visual recognition deficits represent genuine AI limitations rather than assessment inconsistencies. The fact that human experts can reliably evaluate and agree on model performance indicates that the visual interpretation tasks have clear, objective standards that current AI systems fail to meet consistently.

Our histological image interpretation results (50.7-80.1%) align with broader patterns in medical AI visual recognition, falling within the intermediate performance range between general and highly specialized models. Specialized deep learning models demonstrate much more superior performance compared to general vision-language models: while GPT-4 Vision achieved 56.9% accuracy on general medical imaging questions [7], the task-specific deep learning models achieved 80–100% performance across various medical imaging tasks (CT, MRI, X-rays angiograms), including >95% Dice coefficients for liver segmentation, and almost perfect accuracy for cancer detection in histopathology images, and radiologist-level performance in lung cancer screening and COVID-19 detection [29]. Our histological results suggest that histology image interpretation represents a challenging domain for non-specialized LLM visual recognition, possibly due to the microscopic scale, subtle morphological differences, and requirement for fine-grained pattern discrimination that demands even more specialized architectural adaptations.

### Educational implications

The observed performance range (50.7-80.1% accuracy) indicates that current multimodal LLMs are not yet reliable as standalone teaching tools for histology image interpretation, as uncritical use at these accuracy levels could reinforce incorrect pattern recognition or propagate diagnostic errors among learners. However, these systems could provide educational value through carefully designed implementation strategies. Instant feedback systems with confidence thresholding could share only high-confidence responses with students while flagging uncertain cases for instructor review, though this would require the development of calibrated uncertainty estimation capabilities that are currently unavailable in commercial LLM interfaces.

Alternatively, human-in-the-loop approaches could position LLM responses as discussion prompts rather than authoritative answers, thereby encouraging students to critically evaluate AI outputs and develop more discriminating judgment. Comparative learning exercises in which students analyze responses from multiple LLMs alongside expert answers could transform current performance limitations into opportunities to teach critical evaluation skills and to understand AI system constraints. As these systems improve, periodic re-evaluation using the benchmarks established in this study will be essential to determine when accuracy reaches thresholds appropriate for broader educational deployment.

### Study limitations

Several technical and methodological limitations must be acknowledged when interpreting these visual recognition performance results. Our evaluation focused exclusively on static image interpretation without considering dynamic visual analysis capabilities, and the dataset from a single institution may limit generalizability across different staining protocols and imaging systems. Additionally, our use of high-quality, standardized images representing normal tissue architecture for educational purposes may not reflect real-world clinical histopathology variability, including tissue preparation artifacts, fixation inconsistencies, pathological specimens with subtle diagnostic features, and the degraded image quality commonly encountered in routine practice.

The 4-point rating scale may not capture subtle differences in visual recognition quality, and we did not systematically control for image complexity factors such as staining intensity or cellular density that could influence performance differently across models.

The study design presents additional constraints, including the use of identical prompts for all models without architecture-specific optimization, potentially underestimating the performance of models that might benefit from tailored prompt engineering. The rapid advancement in multimodal AI architectures means our 2025 findings may not reflect next-generation visual processing capabilities. Furthermore, the black-box nature of neural network architectures limits our understanding of the specific visual features or processing strategies employed by each model, preventing systematic documentation of visual recognition decision pathways.

### Future directions

The substantial performance gaps identified between text and visual processing capabilities in LLMs highlight critical limitations in their visual processing pipelines rather than knowledge deficits, as these models demonstrate strong textual understanding of medical concepts through multiple-choice questions [25]. The core challenge lies in translating visual information from histological images into the internal representations that LLMs can effectively process, suggesting fundamental architectural limitations in how current visual encoders extract and encode morphological features from microscopic imagery [28]. Several concrete approaches warrant investigation to determine whether current gaps reflect fundamental constraints or optimization opportunities. Prompt optimization studies should systematically compare zero-shot, few-shot, and chain-of-thought prompting strategies, including multimodal chain-of-thought approaches where models verbalize visual observations before interpretation. Model-specific calibration via histology-specific fine-tuning, retrieval-augmented generation using domain-knowledge bases, or the integration of specialized computer-vision models could enhance performance. Dynamic visual analysis that sequentially examines multiple image regions or magnification levels may more closely approximate iterative visual processing strategies. Confidence scoring integration would enable models to identify cases requiring human expert review. Finally, systematic studies that vary image quality, preprocessing, and complexity factors would clarify which factors most affect performance. Testing these approaches would provide future insights beyond the descriptive benchmarks established under the standardized conditions of this study.

## Conclusions

This systematic evaluation reveals fundamental limitations in current multimodal AI visual recognition capabilities. The substantial performance gaps between text-based histology assessment (90.3-92.0% accuracy) and visual interpretation (50.7-80.1% accuracy) reveal that factual knowledge representation does not automatically enable visual pattern recognition, as current architectures demonstrate significantly greater difficulty with spatial reasoning and morphological feature extraction than with text-based medical knowledge processing.

Performance analysis demonstrates that visual processing consistency varies significantly between models (ICC range: 0.562-0.931), contrasting with stable text-based performance reported in prior work. The moderate performance levels across all models indicate systemic constraints in visual processing capabilities rather than model-specific limitations, pointing to fundamental challenges in current multimodal AI development.

These findings establish clear technical benchmarks for measuring progress in multimodal AI development and emphasize the critical need for specialized architectural innovations rather than incremental improvements. Future developments must prioritize enhanced visual processing capabilities, improved cross-modal knowledge transfer mechanisms, and domain-specific training approaches before multimodal AI systems can achieve reliable performance in visually dependent medical applications.

## Supporting information

S1 File Standardized, detailed rubrics for the evaluation criteria of LLM answers. (DOCX)

S2 File Accuracy values for answers of different LLMs. (XLSX)

S3 File Raw data. (CSV)
