# Supplementary material for: Visual recognition limitations in multimodal large language models: A comparative analysis of histological image interpretation
Source: PLOS Digit Health. 2026 Mar 19;5(3):e0001306. doi: 10.1371/journal.pdig.0001306 (PMC13001936; doi:10.1371/journal.pdig.0001306)
Supplement: S1 File — (DOCX) [file pdig.0001306.s001.docx]

Three faculty members holding professorial rank, each with more than 15 years of teaching experience, served as independent evaluators. These experts graded responses independently, without knowing the LLM's identity or other experts' grades, using a 4-point scale: 1 = Poor (Incorrect), 2 = Fair (Partially correct), 3 = Good (Correct), and 4 = Excellent (Absolutely correct). Standardized, detailed rubrics were developed for each question to ensure consistent evaluation criteria of LLM answers:

| Criteria | 1 - Poor/Incorrect | 2 -Fair/Partially correct | 3 - Good/Correct | 4 – Excellent / Absolutely correct |
| --- | --- | --- | --- | --- |
| Q1: Tissue /Organ Identification | Incorrect tissue identification or failure to provide a meaningful response. | Partially correct tissue identification with notable errors in classification or terminology. | Correctly identifies tissue type with minor terminology issues or slight location uncertainty. | Correctly identifies tissue type and anatomical location with appropriate histological terminology. |
| Q2: Structure Identification | Incorrect structure identification or inadequate response. | Partially correct identification with significant descriptive gaps or terminology errors. | Correctly identifies structure with minor descriptive omissions or terminology variations. | Accurately identifies marked structure with precise anatomical terminology and relevant morphological details. |
| Q3: Functional Analysis | Incorrect or inadequate functional description. | Partially accurate functional description with notable gaps or misconceptions. | Accurately describes primary function with minor omissions in secondary functions or context. | Provides a comprehensive and accurate functional description with appropriate physiological context. |
